# Sialylation of IgG inhibits the formation of galactose-deficient IgA1-containing immune complexes and protects mesangial cells from injury in IgA nephropathy

**DOI:** 10.1186/s12882-021-02657-8

**Published:** 2022-01-11

**Authors:** Youxia Liu, Hongfen Li, Huyan Yu, Fanghao Wang, Junya Jia, Tiekun Yan

**Affiliations:** 1 Department of Nephrology, Tianjin Medical University General Hospital, No. 154, Anshan Road, Heping District, Tianjin, PR China; 2 Department of Nephrology, Yunfu People’s Hospital, Yunfu, Guangdong Province PR China

**Keywords:** IgA nephropathy, Sialylated IgG, Immune complex, Sialylated IgG-IgA1 complex, IL-6, TNF-α, TGF-β

## Abstract

**Background:**

The addition of sialic acid alters IgG from a pro-inflammatory state to an anti-inflammatory state. However, there is a lack of research on the changes of IgG sialylation in IgA nephropathy (IgAN).

**Methods:**

This study included a total of 184 IgAN patients. The sialylated IgG (SA-IgG), IgG-galactose-deficient IgA1 complex (IgG-Gd-IgA1-IC), IL-6, TNF-α, and TGF-β were detected using commercial ELISA kits. SA-IgG, non-sialylated IgG (NSA-IgG), sialylated IgG-IgA1 complex (SA-IgG-IgA1), and non-sialylated IgG-IgA1 complex (NSA-IgG-IgA1) were purified from IgAN patients and healthy controls (HCs).

**Results:**

The mean SA-IgG levels in plasma and B lymphocytes in IgAN patients were significantly higher than those of healthy controls. A positive correlation was found between SA-IgG levels in plasma and B lymphocytes. In vitro, the results showed that the release of IgG-Gd-IgA1-IC was significantly decreased in peripheral blood mononuclear cells (PBMCs) cultured with SA-IgG from both IgAN patients and healthy controls. The proliferation ability and the release of IL-6, TNF-α, and TGF-β in human mesangial cells (HMCs) were measured after stimulating with SA-IgG-IgA1-IC and NSA-IgG-IgA1-IC. The mesangial cell proliferation levels induced by NSA-IgG-IgA1-IC derived from IgAN patients were significantly higher than those caused by SA-IgG-IgA1-IC derived from IgAN patients and healthy controls. Compared with NSA-IgG-IgA1 from healthy controls, IgAN-NSA-IgG-IgA1 could significantly upregulate the expression of IL-6 and TNF-α in mesangial cells. The data showed that there weren’t any significant differences in the levels of IL-6, TNF-α, and TGF-β when treated with IgAN-SA-IgG-IgA1 and HC-NSA-IgG-IgA1.

**Conclusions:**

The present study demonstrated that the sialylation of IgG increased in patients with IgA nephropathy. It exerted an inhibitory effect on the formation of Gd-IgA1-containing immune complexes in PBMCs and the proliferation and inflammation activation in mesangial cells.

**Supplementary Information:**

The online version contains supplementary material available at 10.1186/s12882-021-02657-8.

## Introduction

Immunoglobulin A nephropathy (IgAN) is the most common form of primary glomerulonephritis in the world, with 10% of patients will progress to end-stage kidney disease within 10 years after diagnosis [1, 2]. The galactose-deficient IgA1 (Gd-IgA1) and its antibodies (mainly IgG) tend to form immune complexes (IgG-Gd-IgA1-IC) that subsequently deposits on glomerular mesangium and triggers IgAN as well as drives the progression of disease [3–5].

There is growing evidence that the IgG-Fc N-linked glycans site at asparagine 297 (Asn297) provides a key effect on the effector functions of immunoglobulins [6]. An intravenous immunoglobulin used to treat various autoimmune and inflammatory disease has an anti-inflammatory effect due to IgG-Fc glycans rich in sialic acid [7, 8]. Sialylation of IgG (SA-IgG) is a common mechanism of IgG anti-inflammatory action in vivo. Lack of terminal sialic acids in IgG (NSA-IgG) enhances the proinflammatory activity. Importantly, it has been shown that the administration of engineered sialylation of IgG suppressed kidney pathological injury, decreased blood urea nitrogen levels and prolonged the survival in mouse model of Goodpasture disease [9]. However, there is a lack of research on the changes of IgG sialylation in IgAN.

In present study, we detected the expression of SA-IgG in patients with IgAN and determined the effect of SA-IgG on the production of IgG-Gd-IgA1-IC in peripheral blood mononuclear cells (PBMCs). Furthermore, the synthesis of IL-6, TNF-α, and TGF-β was also been examined in SA-IgG-IgA1 and NSA-IgG-IgA1-stimulated human mesangial cells (HMCs). The study preliminary explored the efficacy of SA-IgG in IgAN.

## Materials and methods

### Sample collection

A total of 184 patients with IgAN diagnosed in Tianjin Medical University General Hospital and 50 age- and gender-matched healthy controls (HC) were included in this study. Written informed consent was obtained from all participant. Histopathological diagnosis of IgAN depends on the presence of dominant IgA1 deposition in the glomerular mesangium. We excluded patients suffering from Henoch-Schönlein purpura and other secondary IgAN. Plasma, clinical and histological information were collected at the time of renal biopsy. The Chronic Kidney Disease Epidemiology Collaboration creatinine (CKD-EPI) Collaboration equation was used to calculate estimated glomerular filtration rate (eGFR) [10]. The renal biopsy findings were evaluated and classified according to Oxford classification (MEST-C; M: mesangial hypercellularity; E: endocapillary hypercellularity; S: segmental glomerulosclerosis; T: tubular atrophy/interstitial fibrosis, and C: crescent) [11].

### Flow cytometry for SA-IgG in B cell-surface

After washing and centrifugation, PBMCs in 100 μl phosphate-buffered saline (PBS) with 1% bovine serum albumin (BSA) (Sigma, USA) were stained with Cyanine5.5 mouse anti-human CD19 (Biolegend, USA), phycoerythrin (PE)-conjugated anti-IgG (Biolegend, USA), and fluorescein isothiocyanate (FITC)-conjugated Sambucus nigra lectin (SNA; VECTOR, USA) for FACS analysis. The result was calculated as the percentage of Cyanine5.5 +, PE + and FITC + cells in the gated Cyanine5.5 +, PE + cells population.

### Isolation of sialylated IgG (SA-IgG), non-sialylated IgG (NSA-IgG), sialylated IgG-IgA1 complex (SA-IgG-IgA1) and non-sialylated IgG-IgA1 complex (NSA-IgG-IgA1)

The total serum immunoglobulin was purified using a protein G affinity column (Nunc, Rochester, USA) as previously reported [12, 13]. Purified IgG was desalted with PD-10 column. Next, the IgG solution was drawn into the prepared sambucus nigra lectin (SNA)-agarose column (VECTOR, USA) to allow the buffer drain by gravity. The dropping liquid was collected to acquire non-sialylated IgG (NSA-IgG). Sialylated IgG (SA-IgG) was eluted by adding the eluting solution (VECTOR, USA) before washing with PBS. Each SA-IgG and NSA-IgG sample was depleted of IgA1 using jacalin agarose. The circulation immune complex (CIC) was purified from the buffer containing NSA-IgG and SA-IgG by affinity chromatography using Jacalin immobilized on agarose (Pierce Chemical Company, USA) and eluted with acidic buffer that was subsequently neutralized. Fractions containing IgG were concentrated by a protein centrifugal filter unit (Millipore, USA). Enrichment of the eluate was verified by sialic acid ELISA kit (ab282912) and western blotting with biotinylated SNA, IgG, and IgA antibodies.

### ELISA assay for SA-IgG in plasma

The level of SA-IgG in plasma was determined using ELISA according the slightly modified procedure (ab100547; Abcam, UK). Briefly, 100 μL of diluted plasma was added into each well and incubated for 2.5-hs at room temperature. After washing, 100 μL diluted biotinylated-SNA (1:50, VECTOR, USA) was added for 1 h. The horseradish peroxidase (HRP)-streptavidin solution was added for 45 minutes, followed by incubation with TMB solution and the stop solution. The optical densities (ODs) were determined at 450 nm with an EL312 Bio-Kinetics microplate reader (Bio-TekInstruments, Winooski, VT).

### Peripheral blood mononuclear cells culture and treatment

Approximately 5 mL peripheral venous blood was collected into ethylenediaminetetraacetic acid (EDTA) anticoagulated tubes. PBMCs were seeded into 12-well plates and maintained in RPMI 1640 medium supplemented with 10% fetal bovine serum and treated in six different ways for 48 hours. Group 1: PBMCs treated with medium control (RPMI 1640 medium without the addition of stimulus); Group 2: medium control without cells; Group 3: PBMCs treated with SA-IgG (50 μg/ml); Group 4: SA-IgG (50 μg/ml) treated medium without cells; Group 5: PBMCs treated with NSA-IgG (50 μg/ml); Group 6: NSA-IgG (50 μg/ml) treated medium without cells. Lastly, after centrifugation, the cell culture supernatant of PBMCs was collected.

### ELISA assay for IgG-Gd-IgA1-IC

The Gd-IgA1-IgG-IC level in cell supernatants of PBMCs was determined using an ELISA kit according to a slightly modified procedure (IBL, Japan). In short, Gd-IgA1-specific antibodies and KM55 were immobilized on 96-well ELISA plates. It was followed by adding 100 μL cell supernatant in 6 groups for 2 hours at room temperature. After washing with PBST, the wells were incubated in 1:2000 dilution with HRP conjugated goat anti-human IgG for 1 hour. After washing, the plates were treated with TMB solution and stopped with 2M H_2_SO_4_, and the ODs were determined at 450 nm. The OD value of the medium control group was calibrated as OD450 (Group 1)-OD450 (Group 2). Similarly, the OD values of SA-IgG and NSA-IgG treated groups were calibrated as OD450 (Group 3)-OD450 (Group 4) and OD450 (Group 5)-OD450 (Group 6), respectively.

### Human glomerular mesangial cells (HMCs) culture and treatment

HMCs were purchased from BeNa Culture Clooection (Beijing, China). HMCs were serum-starved before stimulation experiments. Cells were stimulated with human circulating SA-IgG-IgA1 (20 μg/ml) and NSA-IgG-IgA1 (20 μg/ml) -containing immune complexes from IgAN patients and healthy controls (HCs) for 24 hours to detect the changes in proliferation and the levels of cytokines.

### ELISA assay for Gd-IgA1, IL-6, TNF-α and TGF-β

The Gd-IgA1 (IBL, Japan) levels in plasma and the IL-6 (R&D-Valukine ELISA kit, VAL102, USA), TNF-α (R&D-Valukine ELISA kit, VAL105, USA), and TGF-β (R&D-Valukine ELISA kit, VAL127, USA) levels in cell supernatants of HMCs were measured using commercial ELISA kits according to the manufacturer’s instructions.

### Cell viability detected with CCK-8 assay

Cell viability was assessed using a cell counting kit-8 (CCK-8). HMCs were seeded in 96-well plates and stimulated with circulating SA-IgG-IgA1 (20 μg/ml) and NSA-IgG-IgA1 (20 μg/ml). After 24 hours incubation, we added 10 μL of CCK8 solution into each well, which was incubated for 1 h at 37°C continually. The absorbance was measured at a wavelength of 450 nm.

### Western blot

In vitro prepared SA-IgG and NSA-IgG boiled with 5×SDS loading buffer for 10 min. The samples were separated on 10% SDS-PAGE gels and transferred onto a NC membrane. The membrane was incubated with diluted biotinylated-SNA (1:100, VECTOR, USA) at 4 °C overnight. Subsequently, HRP-streptavidin (1:1000, Abcam, UK) was added to bind SNA. The band was detected by western lightning plus ECL reagent (GE Healthcare, USA).

### Statistical analysis

The data gathered from at least 3 independent experiments were averaged according to different groups. Continuous normal distributed variables of two groups were expressed as the mean ± standard deviation (SD) and compared with the unpaired student’s t test. The values of more than two groups were compared using an analysis of variance test. Nonnormally distributed data were expressed as the median and interquartile range and analyzed with the Mann-Whitney U test. For categorical variables, data were analyzed as percentages and compared with an x^2^ test. Pearson’s correlation was applied to analyze correlations. A 2-tailed *P*-value less than 0.05 was considered statistically significant. Statistical analysis was performed by SPSS 16.0 software.

## Results

### Patients with IgAN had high levels of SA-IgG

**Fig. 1:**
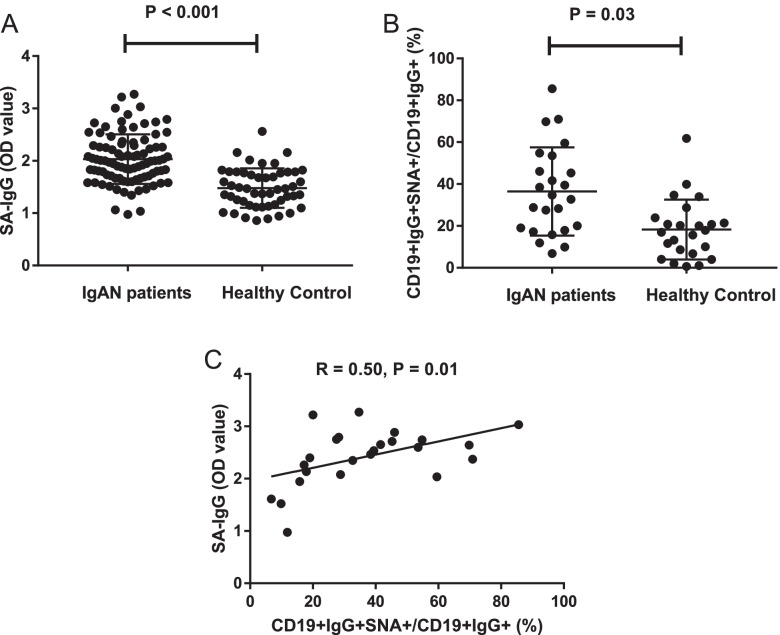
Increased sialylated IgG (SA-IgG) in 184 patients with IgAN. **A** Plasma sialylated IgG levels in 184 patients with IgAN and 50 healthy controls. **B** The percentage of CD19+IgG+SNA+ cells in CD19+IgG+ cells from 24 patients with IgAN and 24 healthy controls. **C** Correlation between SA-IgG levels in plasma and CD19+IgG+ cells

The mean SA-IgG levels in plasma in patients with IgAN were significantly higher than that of healthy controls (2.03 ± 0.47 vs. 1.48 ± 0.38, *P* < 0.001, Fig. 1A). We also compared the levels of SA-IgG in B lymphocytes secreting IgG. The results from flow cytometry revealed the SA-IgG levels in CD19+ cells were increased in patients with IgAN (36.47 ± 21.07 % vs. 18.26 ± 14.28 %, *P* = 0.03, Fig. 1B). There was a positive correlation between SA-IgG level in plasma and B lymphocytes (*r* = 0.50, *P* = 0.01, Fig. 1C).

We further explored the association of plasma SA-IgG with clinical findings and pathological lesions in patients with IgAN. We found that patients with higher SA-IgG levels (OD > 2.02) had significantly lower levels of Gd-IgA1 compared with those patients with lower SA-IgG (OD < 2.02) (Table 1). There was no significant difference in age, gender, SBP, eGFR, and MEST-C percentage between patients with higher and lower levels of SA-IgG (Table 1).

**Table 1:** The baseline data for IgAN patients with lower and higher sialylated IgG (SA-IgG) levels

Characters	Mean ± SD or n (%)		*P*
	Lower SA-IgG (OD > 2.02)	Higher SA-IgG (< 2.02)	
Gender (M/F)	46 (50)/46 (50)	43 (52)/39 (48)	0.75
Age (mean ± SD, year)	39.3±13.36	40.12±13.36	0.68
SBP (mmHg)	131.52±18.21	132.59±16.73	0.68
DBP (mmHg)	80.58±11.51	82.68±11.51	0.22
BMI (kg/m2)	24.57±4.02	24.88±3.86	0.62
Hemoglobin (g/L)	133.64±16.74	129.16±18.74	0.09
Serum albumin (g/L)	36.78±5.19	36.90±4.85	0.87
Triglyceride (mmol/L)	2.06±1.66	2.25±2.44	0.57
Serum creatinine (μmol/L)	85.98±36.55	89.95±54.73	0.61
eGFR (mL/min/1.73 m2)	92.22±26.91	93.08±37.63	0.86
Uric acid (μmol/L)	368.7±101.28	377.77±109.87	0.56
Serum IgA (mg/dL)	408.24±270.22	407.48±315.29	0.99
Serum IgG (mg/dL)	994.31±356.51	1066.39±347.24	0.18
Serum IgM (mg/dL)	105.19±43.75	118.53±56.22	0.09
Serum IgE (mg/dL)	88.24±181.58	132.19±471.25	0.44
Serum C3 (mg/dL)	89.96±17.5	90.43±17.93	0.86
Serum C4 (mg/dL)	22.22±6.11	24.55±8.64	0.06
Proteinuria (mg/24 h)	1853.82±1942.8	1644.17±1340.79	0.4
Urine RBC (/HP)	37.18±64.36	54.38±168.04	0.36
Gd-IgA1 (μg/ml)	12.23±6.94	10.14±5.06	0.02
Oxford classification			
M score (M0/M1)	5 (6)/77(94)	4(5)/78(95)	0.73
E score (E0/E1)	51(62)/31 (38)	46(56)/36 (44)	0.43
S score (S0/S1)	38 (46)/44 (54)	35(43)/47 (57)	0.64
T score (T0/T1/T2)	37(45)/39 (48)/6 (7)	35 (43)/40 (49)/8(8)	0.84
C score (C0/C1/C2)	21 (26)/49 (60)/12 (14)	20(24)/44 (54)/18 (22)	0.48

### Verification of successful preparation of SA-IgG, NSA-IgG and their complex of SA-IgG-IgA1 and NSA-IgG-IgA1

**Fig. 2:**
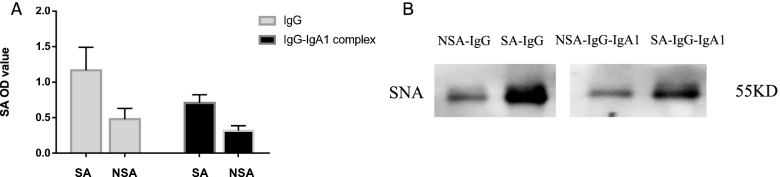
Verification of successful preparation of SA-IgG, NSA-IgG and complexes. **A** Sialic acid status of IgG and IgG-IgA1 complex were detected by sialic acid ELISA kit. SA-IgG and its complex showed high levels of sialic acid . In contrary, NSA-IgG and its complex showed low levels of sialic acid. **B** Existence of SA-IgG, NSA-IgG and complexes were tested by Western blot

We used two methods to detect the sialic acid status of in vitro prepared SA-IgG, NSA-IgG and the complex of SA-IgG-IgA1 and NSA-IgG-IgA1. Compared with SA-IgG, NSA-IgG showed less binding to SNA and lower OD values (Fig. 2A), which indicated that SA-IgG presented with increased sialic acid content and NSA-IgG presented with decreased sialic acid content. Western blot was used to verify the existence of sialic acid in IgG and complexes (Fig. 2B), implied successful in vitro preparation of SA- and NSA- IgG and complexes.

### SA-IgG downregulated IgG-Gd-IgA1-IC secretion in PBMC

**Fig. 3:**
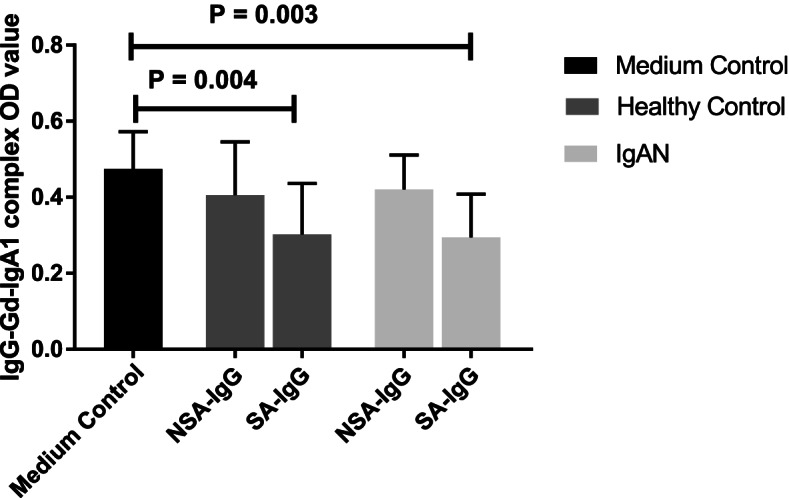
The expression of IgG-Gd-IgA1 complex after sialylated IgG (SA-IgG) stimulation in supernatant of peripheral blood mononuclear cells

As depicted in Fig. 3, the release of IgG-Gd-IgA1-IC were significantly decreased in PBMCs cultured with SA-IgG from healthy controls (0.47 ± 0.09 vs. 0.30 ± 0.13, *P* = 0.004) and from patients with IgAN (0.47 ± 0.09 vs. 0.29 ± 0.11, *P* = 0.003). The findings implied SA-IgG inhibited the combination of IgG autoantibody with Gd-IgA1.

### Effect of SA-IgG-IgA1-IC and NSA-IgG-IgA1-IC on cell proliferation and synthesis of IL-6, TNF-α and TGF-β in HMCs

**Fig. 4:**
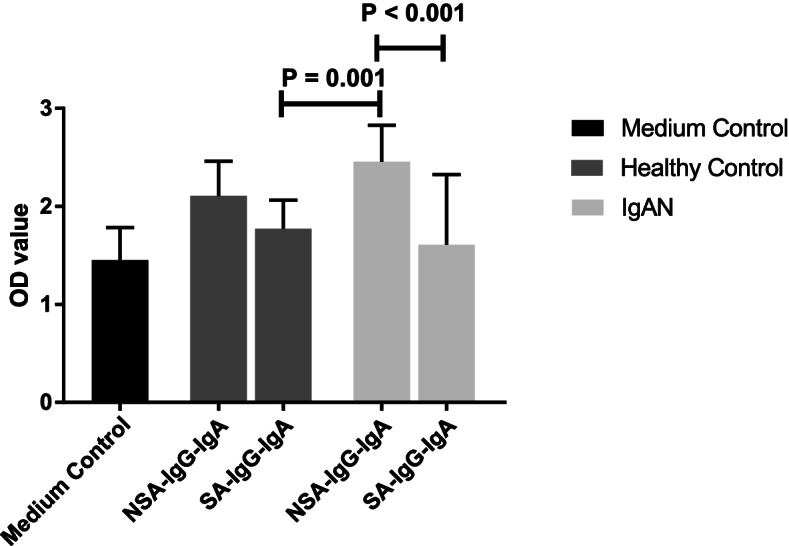
Assay for proliferation after sialylated IgG (SA-IgG) stimulation in supernatant of peripheral blood mononuclear cells

The proliferation ability of HMCs was measured by CCK-8 after stimulated by SA-IgG-IgA1-IC and NSA-IgG-IgA1-IC from IgAN patients and healthy controls. The results showed the levels of mesangial cells proliferation induced by NSA-IgG-IgA1-IC derived from IgAN patients were significantly higher than those caused by SA-IgG-IgA1-IC derived from healthy controls (2.46 ± 0.37 vs. 1.77 ± 0.29, *P* = 0.004) and IgAN patients (2.46 ± 0.37 vs. 1.61 ± 0.72, *P* = 0.004), which was shown in Fig. 4. The degree of HMCs proliferation was higher in the IgAN-NSA-IgG-IgA1-IC group than that in the HC-NSA-IgG-IgA1-IC group, however, the difference didn’t reach statistical significance (2.46 ± 0.37 vs. 2.11 ± 0.35, *P* = 0.07, Fig. 4). The results implied there was no significant increase in proliferation in HMCs when exposed to SA-IgG-IgA1-IC from IgAN patients.

**Fig. 5:**
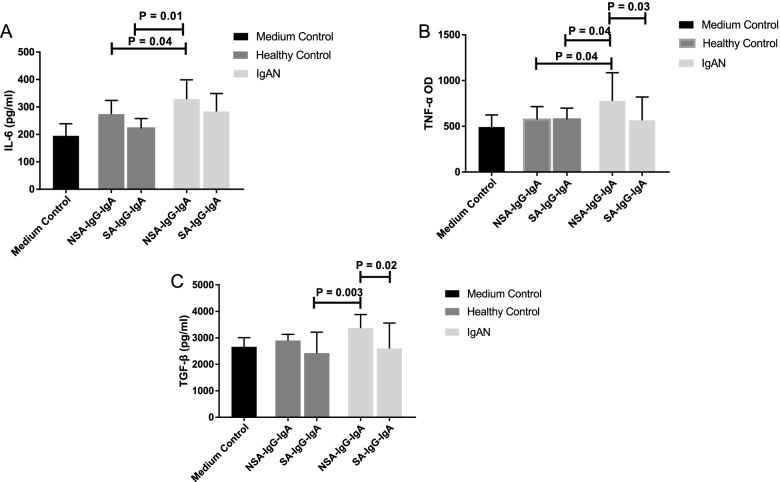
The expression of IL-6 (**A**), TNF-α (**B**) and TGF-β (**C**) in supernatant of mesangial cells after SA-IgG-IgA1 and NSA-IgG-IgA1 stimulation from patients of IgAN and healthy controls

We compared the release of IL-6, TNF-α, and TGF-β in HMCs cultured with SA-IgG-IgA1 and NSA-IgG-IgA1. Our results showed that compared to NSA-IgG-IgA1 from healthy controls, IgAN-NSA-IgG-IgA1 significantly upregulated the expression of IL-6 and TNF-α in mesangial cells (IL-6: 328.12 ± 70.96 pg/ml vs. 262.89 ± 34.02 pg/ml, *p* = 0.04, Fig. 5A; TNF-α: 777.58 ± 307.61 pg/ml vs. 573.94 ± 142.14 pg/ml; *p* = 0.04, Fig. 5B). There was a downward trend in the TGF-β level (IgAN vs. HC 337.01 ± 509.54 pg/ml vs. 301.46 ± 244.57 pg/ml, *P* = 0.06, Fig. 5C). The data also showed that there were no significant differences in the levels of IL-6, TNF-α, and TGF-β when treated with IgAN-SA-IgG-IgA1 and HC-NSA-IgG-IgA1, implying that compared to SA-IgG-IgA1 from IgAN, NSA-IgG-IgA1 could induce the activation of human mesangial cells.

## Discussion

Recently, there is growing evidence that sialylated IgG, a minor component of serum IgG antibodies, is a common mechanism of IgG anti-inflammatory action in immuno-inflammatory diseases [14–16]. Sialylation of pathogenic antibodies in vivo attenuates autoimmune disease [17, 18]. However, there is a lack of research on SA-IgG in IgAN. To the best of our knowledge, this is the first report of plasma SA-IgG elevated in patients with IgAN. In vitro studies, the data provided evidence that SA-IgG could inhibit the combination of IgG autoantibodies with Gd-IgA1 in PBMCs. Moreover, compared with NSA-IgG-IgA1, SA-IgG-IgA1 from IgAN patients resulted in decreased efficacy in inducing the production of IL-6, TNF-α, and TGF-β in HMCs. These sialylated glycan traits of IgG displayed a protective role in IgAN.

Reportedly, the development of many autoimmune diseases is associated with a change of IgG sialylation. Studies involving rheumatoid arthritis (RA) patients revealed decreased sialylation of IgG when compared with healthy controls [19, 20]. Additionally, decreased sialylation of IgG autoantibodies is correlated with the disease activity, duration, and progression of RA [21]. Vuckovic F et al. found that major sialylated glycans of IgG were significantly decreased in patients with SLE [22]. In this study, we found significantly increased plasma levels of SA-IgG in a cohort of IgAN patients. Moreover, we also observed a much higher percentage of CD19+IgG+SNA positive cells in IgAN. We speculate that SA-IgG was actively upregulated in an effort to protect the body from damage. Indeed, in our previous study, we found that ST6GAL1 sialyltransferase, an enzyme that added α2-6-linked sialic acids to the termini of N-glycans of IgG, was increased in IgAN [23]. Besides, the elevated ST6GAL1 level was associated with slower kidney disease progression. Also, compared to the control, purified SA-IgG-treated PBMCs exhibited a significant reduction in the expression of IL-6 and TNF-α [13].

Blood elevated CIC was composed of galactose-deficient IgA1 complexed with antiglycan antibodies, some of which activate glomerular injury. Dana et al. showed that glomerular immune deposits of IgAN patients were enriched for IgG autoantibodies specific for Gd-IgA1 [24]. According to our results, the addition of sialylated IgG could inhibit the IgG-Gd-IgA1 levels in the supernatant of peripheral blood mononuclear cells. Additionally, the purified NSA-IgG-IgA1 from IgAN could upregulate the proliferation of HMCs and the secretion of inflammation cytokines, implying that the purified NSA-IgG-IgA1 in vitro could activate HMCs, similar to circulating CIC in vivo. However, the proliferation levels and inflammatory cytokines secreted from HMCs challenged by SA-IgG-IgA1 from IgAN patients were comparable with NSA-IgG-IgA1 from healthy controls. The comparison indicated that kidney injury might be caused by the desialylated IgG in the complexes, while sialylated IgG sialylation reduced the binding of IgG antibodies to Gd-IgA1 to form the IgG-Gd-IgA1 complex. Meanwhile, the sialylated IgG-IgA1-IC further decreased the efficacy to induce the activation of HMCs. In previous studies, the addition of sialic acid alters IgG from a pro-inflammatory to an anti-inflammatory state. Inflammatory cytokines are known to be involved in the production of Gd-IgA1 and IgG-IgA IC in IgAN [25–27]. With the knowledge that IgG sialylation affects the anti-inflammatory activity of IgG, this may be an indication that the degree of SA-IgG is a biomarker and a functional effector of delaying disease progression. The 2020 KDIGO suggests that the management of IgAN is primarily focused on supportive care and immunosuppressive-based therapies. However, currently, there aren’t any effective, safe, and disease-specific therapies [28]. As a different path of study, investigating sialic acid changes in IgG is supposed to a be a potential strategy to provide added value regarding diagnosis and therapy of IgAN.

### Limitations

The present study has several limitations. Firstly, due to the small sample size and fewer endpoints, the authors did not evaluate the prognostic value of SA-IgG in IgAN patients. Secondly, because of the limited quantity of SA-IgG-IgA1-complexes isolated from circulation, we did not have sufficient CICs to explore whether this protective action was in a concentration-dependent manner. Thirdly, the mechanism of anti-inflammatory activities of SA-IgG was not fully understood. Therefore, in the future, additional longitudinal studies involving more diverse patient cohorts are required to validate the protective role of SA-IgG in IgAN and investigate the suppressive role of sialylated IgG during IgAN.

## Conclusion

Our study demonstrated that sialylation of IgG increased in patients with IgA nephropathy. More specifically, it exerted an inhibitory effect on the formation of Gd-IgA1-containing immune complexes in PBMCs and the proliferation and inflammation in mesangial cells. The present study was the first study to highlight the important role of sialylated IgG in IgAN, which provided a potential therapeutic target for the treatment of IgAN.

## Supplementary Information

**Additional file 1.**

## Data Availability

Raw data used during the current study are available from the corresponding author on reasonable request for non-commercial use.
