# Supplementary material for: Sialylation of IgG inhibits the formation of galactose-deficient IgA1-containing immune complexes and protects mesangial cells from injury in IgA nephropathy
Source: BMC Nephrol. 2022 Jan 11;23:25. doi: 10.1186/s12882-021-02657-8 (PMC8751338; doi:10.1186/s12882-021-02657-8)

Sialylation of IgG inhibits the formation of galactose-deficient IgA1-containing immune complexes and protects mesangial cells from injury in IgA nephropathy

Authors:

Youxia Liu^1^, Hongfen Li^1^, Huyan Yu^2^, Fanghao Wang^1^, Junya Jia^1^ and Tiekun Yan^1^

Affiliations:

1. Department of Nephrology, Tianjin Medical University General Hospital, Tianjin, PR China

2. Department of Nephrology, Yunfu People’s Hospital, Yunfu, Guangdong province, PR China

Running head: IgG Sialylation is IgA nephropathy

Corresponding:

Youxia Liu and Junya Jia

Department of Nephrology, Tianjin Medical University General Hospital

No. 154, Anshan Road, Heping District, Tianjin, China

Email: [5liuyouxia@163.com](mailto:5liuyouxia@163.com) or [jiajunya@126.com](mailto:jiajunya@126.com)

Telephone: 86-20-60362590

Word count of abstract: 248

Word count of text: 3300

Tables: 3

Figures: 5


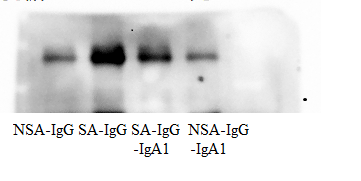

Supplement: Supplementary file 1 — Additional file 1. [file 12882_2021_2657_MOESM1_ESM.docx]
